# Meta-analysis of MMP-9 levels in the serum of patients with epilepsy

**DOI:** 10.3389/fnins.2024.1296876

**Published:** 2024-02-21

**Authors:** Qin Wang, Zehua Lin, Chunyuan Yao, Jinwen Liu, Jiangwei Chen, Limei Diao

**Affiliations:** ^1^Graduate School of First Clinical Medicine College, Guangxi University of Chinese Medicine, Nanning, Guangxi, China; ^2^Department of Neurology, The First Affiliated Hospital of Guangxi University of Chinese Medicine, Guangxi, Nanning, China; ^3^Zhongnan Hospital of Wuhan University, Wuhan, Hubei, China

**Keywords:** MMP-9, epilepsy, meta-analysis, brain, seizure

## Abstract

**Background:**

Epilepsy’s pathogenesis and progression are significantly influenced by neuroinflammation, blood–brain barrier function, and synaptic remodeling function. Matrix metalloproteinase 9 (MMP-9), as a critical factor, may contribute to the development of epilepsy through one or more of the above-mentioned pathways. This study aims to evaluate and quantify the correlation between MMP-9 levels and epilepsy.

**Methods:**

We conducted a comprehensive search of Embase, Web of Science, PubMed, Cochrane Library, WanFang DATA, VIP, and the CNKI to identify studies that investigate the potential association between MMP-9 and epilepsy. The data were independently extracted by two researchers and assessed for quality using the Cochrane Collaboration tool. The extracted data were analyzed using Stata 15 and Review Manager 5.4. The study protocol was registered prospectively at PROSPERO, ID: CRD42023468493.

**Results:**

Thirteen studies with a total of 756 patients and 611 matched controls met the inclusion criteria. Eight of these studies reported total serum MMP-9 levels, and the other five studies were used for a further subgroup analysis. The meta-analysis indicated that the serum MMP-9 level was higher in epilepsy patients (SMD = 4.18, 95% confidence interval = 2.18–6.17, *p* < 0.00001) compared with that in the control group. Publication bias was not detected according to Begg’s test. The subgroup analysis of country indicated that the epilepsy patients in China, Poland, and Egypt had higher levels of serum MMP-9 than the control group, with the increase being more pronounced in Egypt. The subgroup analysis of the age category demonstrated that the serum MMP-9 levels of the adult patients with epilepsy were significantly higher than those of the matched controls. However, the serum MMP-9 levels did not significantly differ in children with epilepsy. The subgroup analysis of the seizure types demonstrated substantial difference in the MMP-9 levels between patients of seizure-free epilepsy (patients who have been seizure-free for at least 7 days) and the control group. Meanwhile, the serum MMP-9 level in patients with epileptic seizures was significantly higher than that in the control group. The subgroup analysis based on seizure duration in patients showed that the serum MMP-9 levels at 1–3, 24, and 72 h after seizure did not exhibit significant differences between female and male patients with epilepsy when compared with the control group. The serum MMP-9 levels at 1–3 and 24 h were significantly higher than those of the matched controls. Nevertheless, the serum MMP-9 level at 72 h was not significantly different from that in the control group.

**Conclusion:**

This meta-analysis presents the first comprehensive summary of the connection between serum MMP-9 level and epilepsy. The MMP-9 levels in epilepsy patients are elevated. Large-scale studies with a high level of evidence are necessary to determine the exact relationship between MMP-9 and epilepsy.

## Highlights

- This work is the first meta-analysis to investigate the association between serum MMP-9 level and epilepsy.
- The serum level of MMP-9 is elevated in patients with epilepsy, particularly in adults.
- The serum level of MMP-9 may be related to seizure duration, independent of sex.
- The serum level of MMP-9 could potentially serve as a biomarker for epilepsy.

## 1 Introduction

Epilepsy is a chronic recurrent transient brain dysfunction syndrome characterized by abnormal discharges of neurons in the brain (Diao et al., 2015). This condition is listed as one of the five major neurological disorders that WHO focuses on preventing and treating. At present, approximately 70 million people have epilepsy worldwide (GBD 2015 Neurological Disorders Collaborator Group, 2017), the incidence of epilepsy in developing countries is as high as 120,000/year (Hirose, 2013), and nearly 9 million epilepsy patients live in China (Zhen, 2014).

Neuroinflammation, blood–brain barrier (BBB) dysfunction, and altered synaptic remodeling function are potential contributors to seizures. Endogenous and exogenous pathogens can enter the brain through the damaged BBB, resulting in a local inflammatory response that causes leukocytes to flow across the BBB into the brain, stimulating neurons in the brain, causing abnormal excitation of neuronal synapses and altered synaptic remodeling function, leading to seizures (Swann and Hablitz, 2000; Fabene et al., 2008, 2013). Accordingly, BBB dysfunction interacts with neuroinflammation in a vicious cycle that can further increase the BBB permeability (Vezzani et al., 2008; Frigerio et al., 2012).

Matrix metalloproteinases (MMPs) are a class of zinc atom-dependent endopeptidases that exist as zymogens, and extracellularly activated MMPs can selectively degrade a variety of extracellular matrix components. MMP 9 (MMP-9) is one of the more important MMPs, and its important functions in the central nervous system are related to its involvement in neuroinflammation, BBB function, and synaptic plasticity (Yin et al., 2011; Bronisz and Kurkowska-Jastrzębska, 2016). Under physiological conditions, MMP-9 is mainly produced by neurons, and glial cells also produce small amounts of MMP-9 (Michaluk and Kaczmarek, 2007; Yin et al., 2011), which is involved in neuronal plasticity (Michaluk and Kaczmarek, 2007; Dong et al., 2009). However, during seizures, increased cytokine production by circulating immune and neuroglial cells stimulates the activation of metalloproteinases. Consequently, upregulated MMP-9 degrades many extracellular matrix molecules to disrupt the BBB. The increased MMP-9 can also rearrange the molecular structure of peripheral cells and cellular matrix, resulting in synaptic remodeling, which can lead to epilepsy (Bronisz et al., 2022).

Animal experiments suggest that elevated MMP-9 may be closely associated with epileptogenesis, leading to neuronal death, abnormal synaptic plasticity, and neuroinflammation. For instance, glutamate released during epileptic seizures in rats leads to elevated levels of MMP-9 protein expression and activity, resulting in BBB leakage (Rempe et al., 2018). However, direct experimental evidence linking serum MMP-9 to neurological disorders such as epilepsy is still lacking. Several clinical studies have observed a potential association between changes in serum MMP-9 levels and epilepsy. The specific epileptogenic mechanisms of serum MMP-9 are not yet well understood, including its relationship with cytokines and inflammatory response mediators and its interaction mechanisms (Yin et al., 2011). This meta-analysis aims to provide useful evidence for the continuous and in-depth study of serum MMP-9 in epilepsy in the future.

## 2 Materials and methods

This systematic review followed the Preferred Reporting Items for Systematic Reviews and Meta-Analyses guidelines.

### 2.1 Search strategy and selection criteria

Two researchers searched Embase, Web of Science, PubMed, Cochrane Library, WanFang DATA, VIP, and CNKI for relevant articles published up to 20 August 2023. PubMed was searched for MeSH terms as follows: (“epilepsy” OR “epilepsies” OR “seizure disorder” OR “seizure disorders”) AND (“MMPs” OR “MMP-9” OR “Matrix Metalloproteinase 9”) OR “Serum Proteins Associated with Blood–Brain Barrier.”

The inclusion criteria consisted of conducting case–control or cohort studies and reporting the serum levels of MMP-9 in control individuals and epilepsy patients. The exclusion criteria included the use of overlapping population or databases, animal studies, case reports, systematic case reports, systematic reviews, comments, letters, and reviews.

### 2.2 Data extraction

Both researchers independently searched for relevant literature by browsing through the titles and abstracts. They then screened the literature to discover any apparent inconsistencies before subsequently reading the full texts. Finally, they re-screened the literature to make final decisions regarding inclusion. If any disagreement arose during the checking and screening of literature, then it could be resolved either through discussion within the research group or by involving a third-party researcher.

The study’s name of primary article, year and country of publication and details on the assay method, specimen type, total number of cases and controls, ages, sex, and MMP-9 concentration were recorded or calculated. The included studies were evaluated by two independent researchers by using the Newcastle–Ottawa Scale (NOS). Studies with a score equal or greater than six were recognized as high-quality.

### 2.3 Statistical analysis

The statistical analyses were conducted using the RevMan 5.4 and Stata 15 software provided by the official website of the Cochrane Collaboration, following the principles of evidence-based medicine. We used the risk ratio and 95% confidence interval (CI) to examine the continuous or dichotomous variables. Furthermore, we tested for statistical heterogeneity using the *I*^2^ statistic. If no heterogeneity exists between the results of the studies (*p* > 0.1, *I^2^* ≤ 50%), then we used a fixed-effects model in the meta-analysis with the corresponding outcome indicators (Chen et al., 2023). Meanwhile, if heterogeneity existed between the results of the studies, then we used a random-effects model in the meta-analysis. We performed subgroup or regression analyses on factors, such as country, age category, seizure types, and duration of seizures, which could have contributed to the generation of heterogeneity. The leave-one-out method was applied for sensitivity analysis. A funnel plot was used to evaluate publication bias and Begg’s test to determine statistical significance.

When we could not find the cause of the heterogeneity, or the heterogeneity was clearly significantly large, we conducted descriptive analyses.

## 3 Results

### 3.1 Study selection

**Figure 1:**
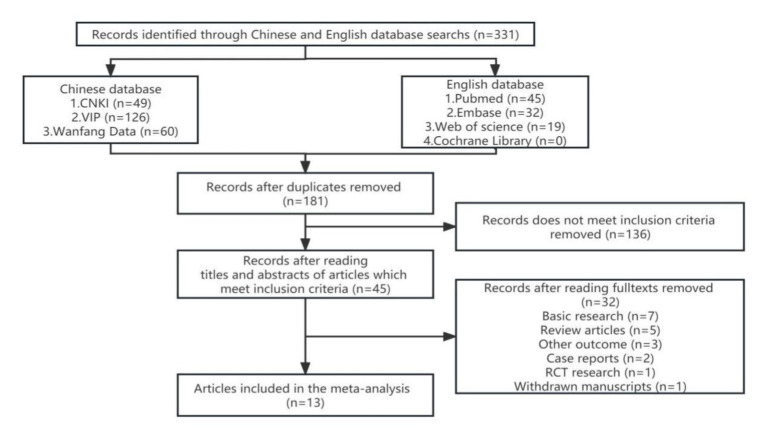
The diagram illustrating the systematic literature search.

We identified 331 references from the initial database search and excluded 181 duplicates. After further review, 136 articles that do not meet inclusion criteria were removed. The titles, abstracts, and full texts were carefully reviewed. The research (*n* = 7), review articles (*n* = 5), other outcome (*n* = 3), case reports (*n* = 2), RCT research (*n* = 1), and withdrawn manuscripts (*n* = 1) were excluded. Finally, thirteen studies were included for quantitative pooling and meta-analysis. Eight of these studies were from the PubMed database, three were from the CNKI database, and two were from the Wanfang database. In total, the thirteen studies included 756 patients and 611 matched controls. The study selection process is summarized in Figure 1. The characteristics of these studies are reported in Table 1. These included studies were published and peer reviewed. All 13 studies were case–control (Zhang and Hao, 2009; Zhang et al., 2013; Cudna et al., 2017; Bronisz et al., 2018; Meguid et al., 2018; El Moneam Abdallah et al., 2019; Wang, 2019; Zhang, 2019; Huang et al., 2020; Li et al., 2020; Tao et al., 2020; Bronisz et al., 2023; Cudna et al., 2023) studies. Enzyme-linked immunoassay is a unified method for MMP-9 detection. The NOS (Stang, 2010) was applied to assess the quality of the selected studies. The results are presented in Table 2.

**Table 1:** Characteristics of the included studies.

Year	Author	Country	Sample sizes (Case/HC)	Gender (Female/Male)	Age category	epilepsy type	Type of MMPs	Sample source	Assay type	Mean age (Case/HC)	Serum MMP-9 Concentrations (^−^x ± s) (Case/HC, ng/mL)
2023	Cudna et al. (2023)	Poland	100 (50/50)	Case:19/31 HC:24/26	Adult	Bilateral tonic–clonic seizures	MMP-9	Serum	ELISA	Case:47 ± 19.7, range (19–85) HC:43.3 ± 16, range (18–88)	1-3 h Case:1447.98 ± 491.54 HC:724.65 ± 295.27 24 h Case:1078.42 ± 402.58 HC:724.65 ± 295.27 72 h Case:863.26 ± 396.32 HC:724.65 ± 295.27 Case:(female n = 19/male n = 31) 1-3 h 1361.95 ± 381.8/1500.72 ± 547.36 24 h 1016.01 ± 360.27/1116.67 ± 427.62 72 h 915 ± 478.18/831.54 ± 341.49 HC:(female n = 24/male n = 26) 736.85 ± 358.84/731.4 ± 228.34
2023	Bronisz et al. (2023)	Poland	200 (100/100)	Case:52/48 HC:52/48	Adult	Epilepsy in the interictal	MMP-9	Serum	ELISA	Case:43.01 ± 1.53, range (19–82) HC:42.16 ± 1.58, range (17–84)	Case:846.66 ± 56.35 HC:533.35 ± 32.89
2020	Huang et al. (2020)	China	247 (127/120)	Case:55/72 HC:51/69	Adult	Epilepsy	MMP-9	SERUM	ELISA	Case:60.4 ± 7.6, range (34–71) HC:59.8 ± 8.0, range (35–70)	Case:1.42 ± 0.39 HC:1.04 ± 0.26
2020	El Moneam Abdallah et al. (2019)	Egypt	60 (30/30)	Case:14/16 HC:14/16	Children	Epilepsy	MMP-9	Serum	ELISA	Case: 8.43 ± 3.33 HC:7.89 ± 2.5	Case:6.45 ± 3.33 HC:2.83 ± 1.23
2020	Tao et al. (2020)	China	151 (101/50)	Case:39/62 HC:22/28	Adult	Epilepsy	MMP-9	Serum	ELISA	Case:38.21 ± 1.59 HC:43.91 ± 1.81	Case:2799.67 ± 69.36 HC:1703.61 ± 67.80 Idiopathic epilepsy n = 43 Case:2882.32 ± 104.14 HC:1703.61 ± 67.80 Symptomatic epilepsy n = 58 Case:2752.44 ± 91.95 HC:1703.61 ± 67.80
2020	Li et al. (2020)	China	94 (64/30)	Case:28/36 HC:13/17	Children	Epilepsy with different frequency and duration	MMP-9	Serum	ELISA	Case:6.3 ± 1.5 HC:6.1 ± 1.4	Case:12.74 ± 1.46 HC:4.52 ± 1.48 Epilepsy non frequent non persistent state n = 27 Case:10.23 ± 1.69 HC:4.52 ± 1.48 Frequent seizures n = 21 Case:12.31 ± 1.21 HC:4.52 ± 1.48 Epileptic status n = 16 Case:14.01 ± 1.75 HC:4.52 ± 1.48 Comprehensive seizure of epilepsy n = 45 Case:13.57 ± 2.36 HC:4.52 ± 1.48 Partial seizure of epilepsy n = 19 Case:13.68 ± 2.41 HC:4.52 ± 1.48
2019	Wang (2019)	China	88 (60/28)	Case:32/28 HC:16/12	Adult	Epilepsy	MMP-9	Serum	ELISA	Case:32.5 ± 13.3 HC:33.0 ± 12.0	Case:118.1 ± 27.6 HC:172.5 ± 63.5
2019	Zhang (2019)	China	90 (60/30)	NA	Children	Idiopathic epilepsy	MMP-9	Serum	ELISA	Epilepsy non frequent non persistent state Case:5.1 ± 0.45 HC:NA Frequent seizures Case:4.2 ± 0.78 HC:NA Epileptic status Case:3.9 ± 0.14 HC:NA	Epilepsy non frequent non persistent state n = 25 Case:9.73 ± 1.57 HC:4.53 ± 0.91 Frequent seizures n = 20 Case:13.17 ± 0.71 HC:4.53 ± 0.91 Epileptic status n = 15 Case:13.94 ± 0.93 HC:4.53 ± 0.91 Comprehensive seizure of epilepsy n = 35 Case:12.34 ± 1.95 HC:4.53 ± 0.91 Partial seizure of epilepsy n = 25 Case:11.32 ± 2.46 HC:4.53 ± 0.91
2018	Meguid et al. (2018)	Egypt	60 (30/30)	NA	Adult	Epilepsy	MMP-9	Serum	ELISA	NA	Case:968.3 ± 661 HC:364.7 ± 223.8
2018	Bronisz et al. (2018)	Poland	88 (44/44)	NA	Adult	Epilepsy	MMP-9	Serum	ELISA	NA	Case:909.7 ± 91.3 HC:634.2 ± 40.8
2017	Cudna et al. (2017)	Poland	86 (43/43)	Case:19/24 HC:19/24	Adult	Generalized tonic–clonic seizure (GTC)	MMP-9	Serum	ELISA	Case:42.8 ± 17.8, range (19–84) HC:43.2 ± 16.8, range (18–88)	Case:(female/male) 1-3 h 1432.88 ± 90.0/1588.1 ± 113.0 24 h 1016.00 ± 82.6/1116.8 ± 82.1 72 h 828.15 ± 73.7/828.44 ± 72.7 HC:(female/male) 738.05 ± 87.2/715.76 ± 48.0
2013	Zhang et al. (2013)	China	71 (31/40)	NA	Adult	Generalized tonic–clonic seizure (GTC)	MMP-9	Serum	ELISA	NA	Case: 4 h 19.57 ± 8.72 24 h 17.69 ± 9.74 60 h 14.62 ± 10.03 7d 13.67 ± 5.53 HC:10.65 ± 1.92
2009	Zhang and Hao (2009)	China	32 (16/16)	Case:7/9 HC:8/8	Children	Epileptic seizure	MMP-9	Serum	ELISA	NA	24 h Case:1.28 ± 0.19 HC:1.02 ± 0.24 72 h Case:1.21 ± 0.22 HC:1.02 ± 0.24

**Table 2:** The quality of the selected studies was assessed using the NOS.

	Studies	Selection	Comparability	Exposure	Total
2023	Cudna et al. (2023)	✫✫✫✫	✫	✫✫	7 stars
2023	Bronisz et al. (2023)	✫✫✫✫	✫✫	✫✫	8 stars
2020	Huang et al. (2020)	✫✫✫	✫✫	✫	6 stars
2020	El Moneam Abdallah et al. (2019)	✫✫✫	✫	✫	5 stars
2020	Tao et al. (2020)	✫✫✫	✫✫✫	✫	7 stars
2020	Li et al. (2020)	✫✫	✫✫	✫✫	6 stars
2019	Wang (2019)	✫✫	✫✫	✫	5 stars
2019	Zhang (2019)	✫✫	✫	✫✫	5 stars
2018	Meguid et al. (2018)	✫✫✫	✫✫	✫✫	7 stars
2018	Bronisz et al. (2018)	✫✫✫✫	✫✫	✫	7 stars
2017	Cudna et al. (2017)	✫✫✫	✫✫	✫	6 stars
2013	Zhang et al. (2013)	✫✫	✫	✫	4 stars
2009	Zhang and Hao (2009)	✫✫	✫	✫	4 stars

### 3.2 Main association between MMP-9 level and epilepsy

**Figure 2:**
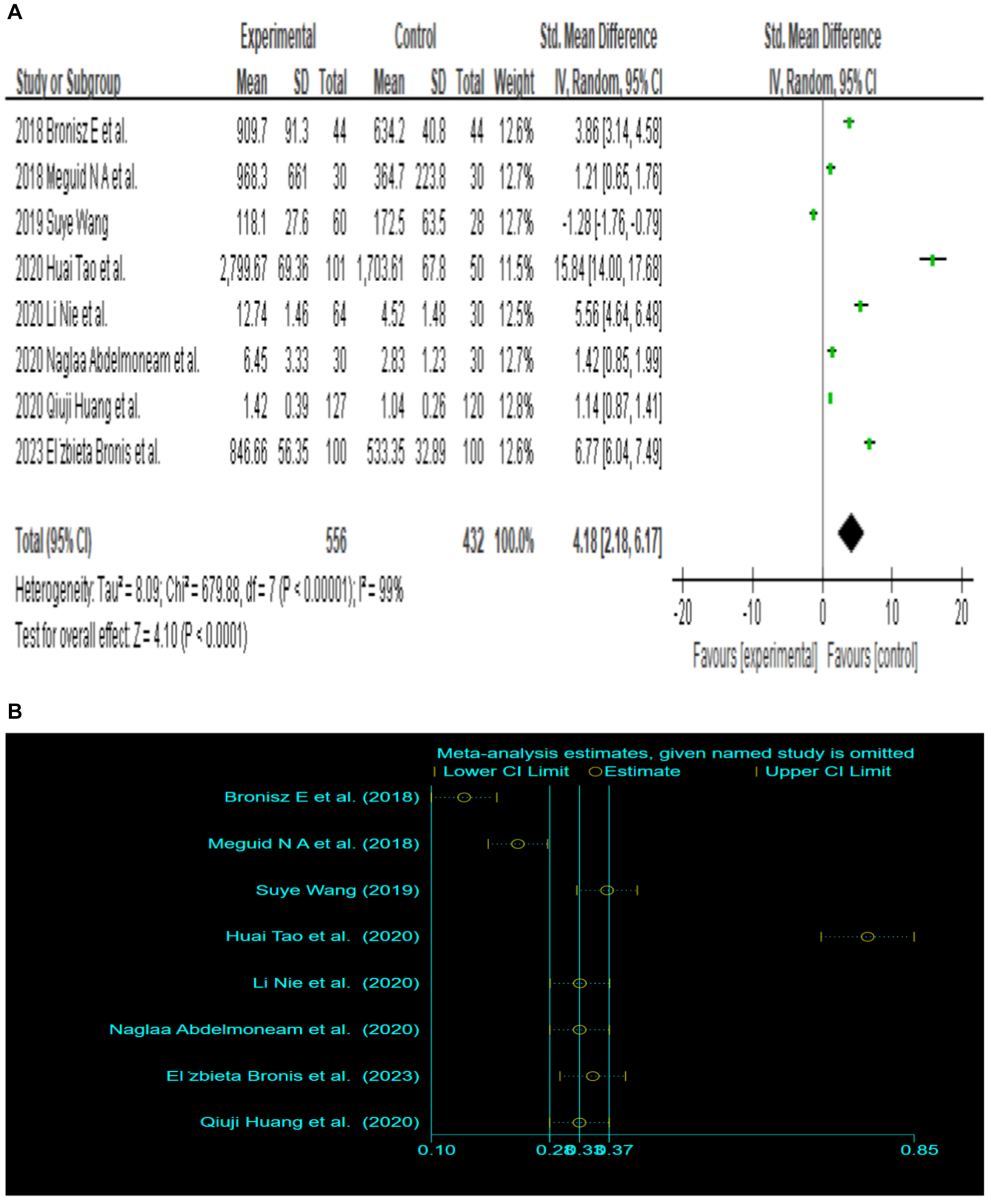

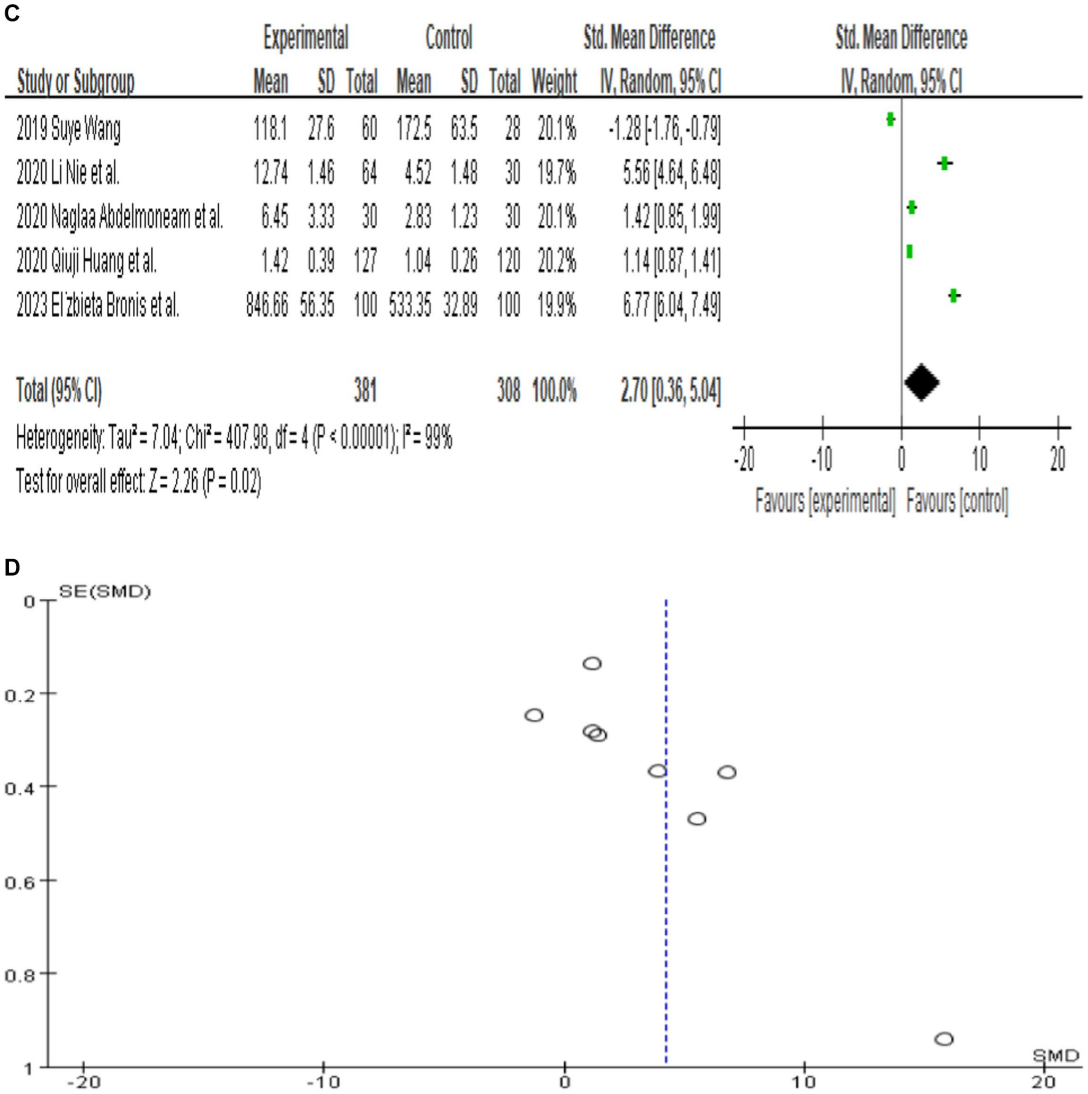
**(A)** Forest plot depicting the relationship between MMP-9 level and epilepsy. **(B)** Sensitivity analysis of this meta-analysis. **(C)** Association between MMP-9 level and epilepsy, following exclusion of the study by Tao et al. (2020), Meguid et al. (2018), and Bronisz et al. (2018). **(D)** A funnel plot to assess publication bias. **(E)** Meta regression indicates that publication year and country are not sources of heterogeneity following exclusion of the study by Huang et al. (2020).

Since only 8 out of the 13 papers had complete total MMP-9 data, we conducted a random-effects meta-analysis of the 8 studies that were included and performed to generate forest plots (Bronisz et al., 2018; Meguid et al., 2018; El Moneam Abdallah et al., 2019; Wang, 2019; Huang et al., 2020; Li et al., 2020; Tao et al., 2020; Bronisz et al., 2023). The remaining 5 papers were used for subgroup analysis. Figure 2A illustrates that the MMP-9 level in patients with epilepsy was significantly higher than that in the matched controls (SMD = 4.18, 95% CI = 2.18–6.17, *p* < 0.00001; Figure 2A). A sensitivity analysis was conducted to evaluate the stability of the results. The studies by Bronisz et al. (2018), Meguid et al. (2018), Tao et al. (2020) had a substantial influence on the results. Such studies significantly extended the effect size, which may have a detrimental effect on the robustness of the meta-analysis results (Figure 2B). After exclusion of the three studies, the results changed (SMD = 2.70, 95% CI = 0.36–5.04, *p* < 0.00001), and the heterogeneity decreased (Figure 2C). Accordingly, the current meta-analysis data are relatively reliable and credible. Visual assessment of a funnel plot did not reveal publishing bias (Figure 2D). Begg’s test was performed on eight studies (Z = 1.35, *p* = 0.315). Given that *p* > 0.05, the results indicated no publication bias. However, the meta-analysis did show severe heterogeneity (df = 4, *p* < 0.0001, *I*^2^ = 99%).

**Figure 3:**
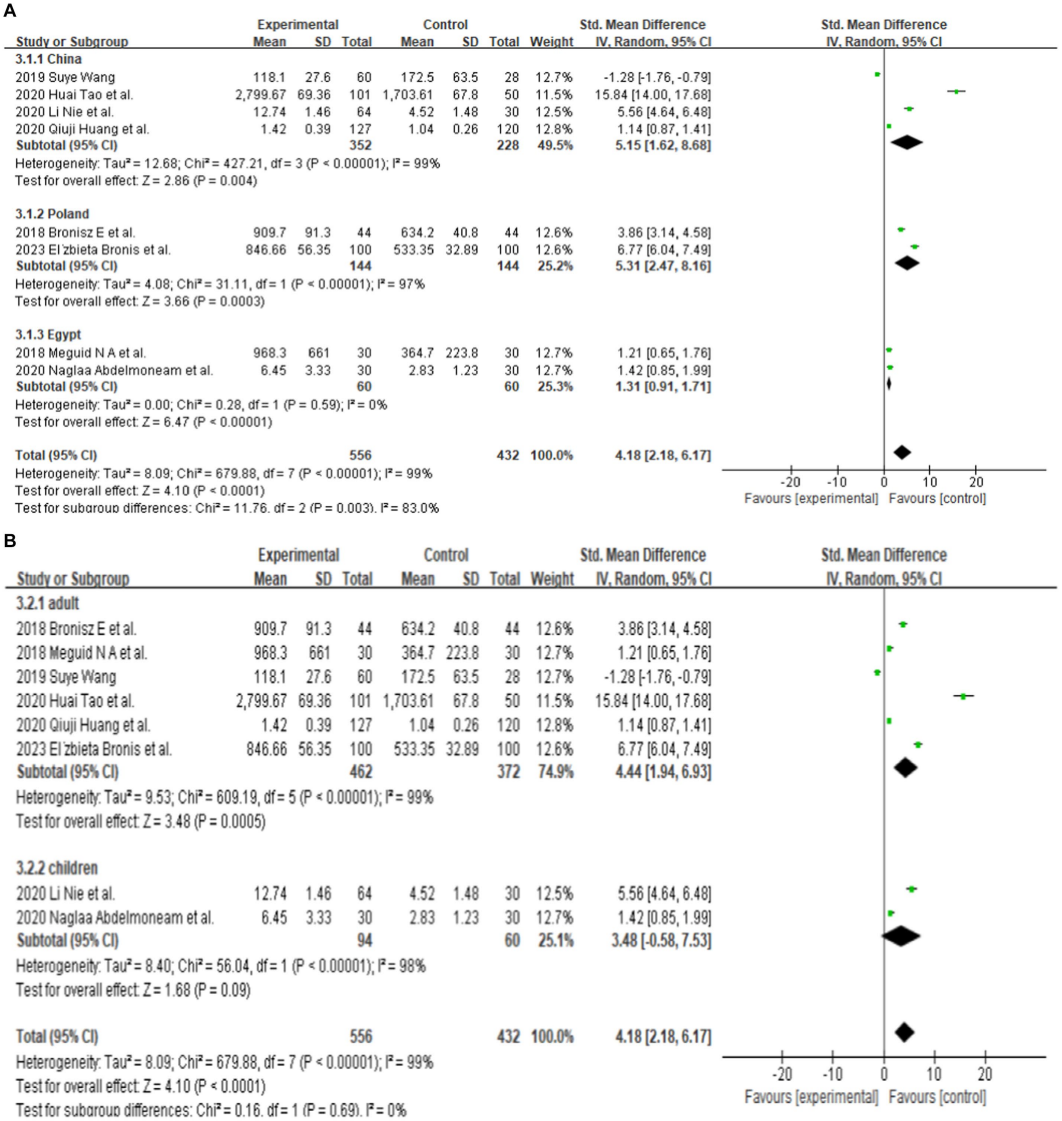

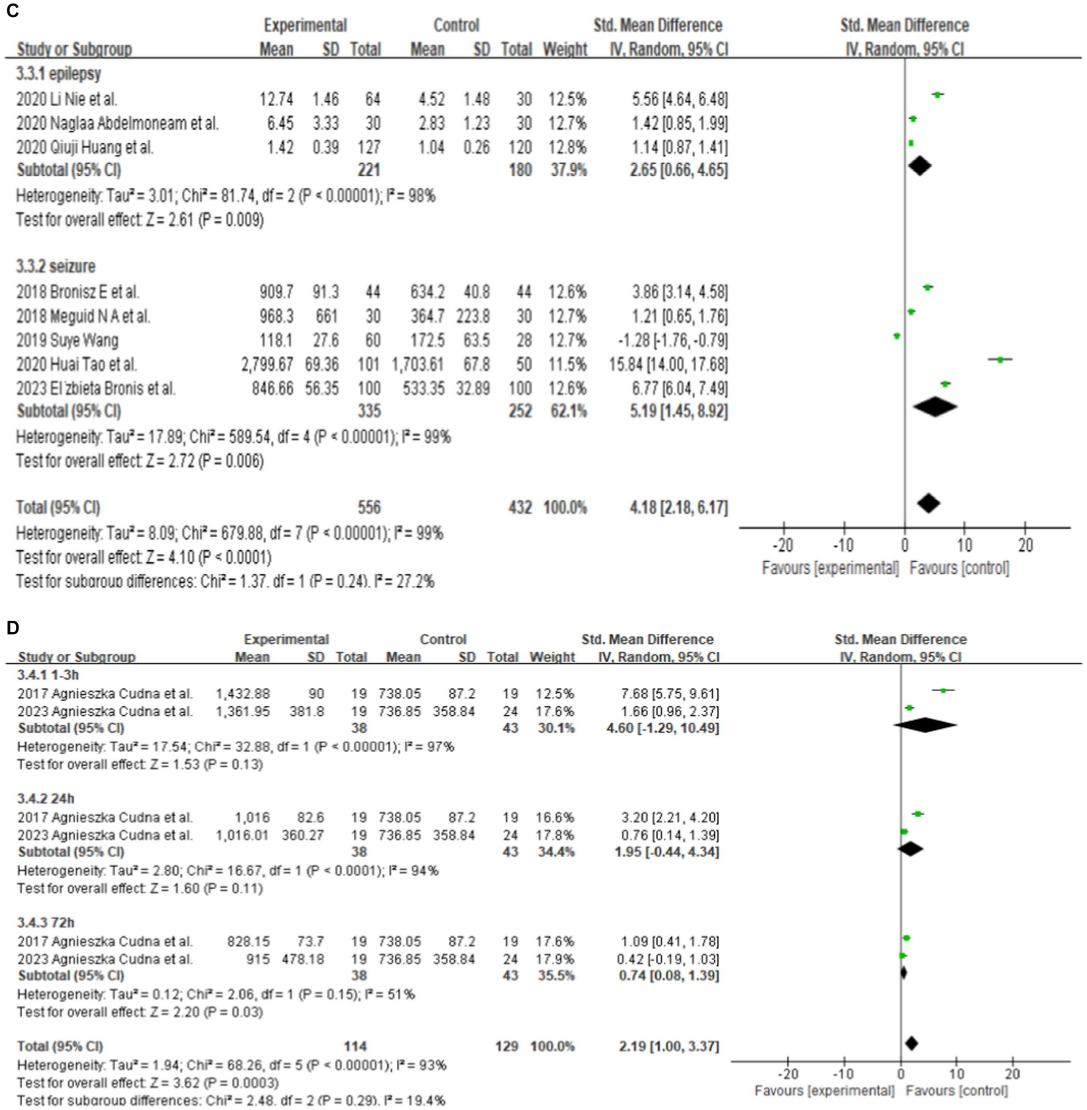

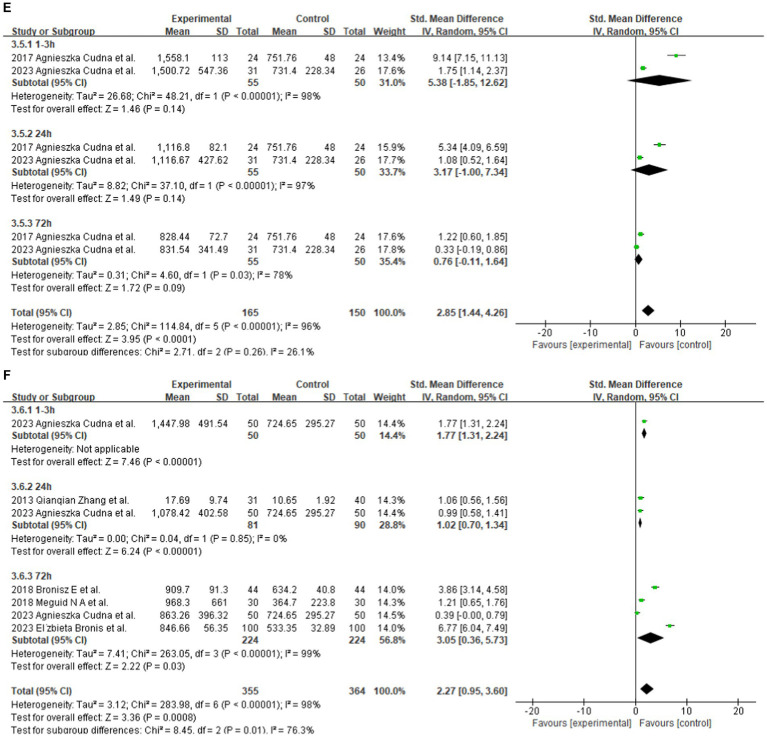
**(A)** Subgroup analysis by country demonstrating the association between serum MMP-9 level and epilepsy. **(B)** Subgroup analysis by age category demonstrating the association between serum MMP-9 level and epilepsy. **(C)** Subgroup analysis by seizure types demonstrating the association between serum MMP-9 level and epilepsy. **(D)** Subgroup analysis by female duration of seizures demonstrating the association between serum MMP-9 level and epilepsy. **(E)** Subgroup analysis by male duration of seizures demonstrating the association between serum MMP-9 level and epilepsy. **(F)** Subgroup analysis by duration of seizures demonstrating the association between serum MMP-9 level and epilepsy.

Further meta-regression analysis (*p* > 0.05) showed that the year of publication and country were not the sources of heterogeneity in these eight papers. It is important to note that the meta-regression analysis was calculated based on the _ES value. Huang et al. (2020) had a value of 0 for _ES and were therefore excluded from the analysis. In the end, seven studies were included in this meta-regression analysis (Figure 2E). And the result of meta-regression analysis is consistent with the results of the subgroup analyses that explored the source of heterogeneity based on country (Figure 3A).

### 3.3 Subgroup analysis by country

Eight studies reported serum MMP-9 levels in epilepsy patients and matched controls. Four studies reported MMP-9 levels in epilepsy patients and matched controls from China. The subgroup analysis of different countries was performed, and random effect models were selected based on the presence of heterogeneity (China: df = 3, *I*^2^ = 99%, *p* < 0.00001; Poland: df = 1, *I*^2^ = 97%, *p* < 0.00001; Egypt: df = 1, *I*^2^ = 0%, *p* = 0.59; Figure 3A). The meta-analysis indicated that the MMP-9 levels in China (SMD = 5.15, 95% CI = 1.62–8.68, *p* = 0.004; Figure 3A), Poland (SMD = 5.31, 95% CI = 2.47–8.16, *p* = 0.0003; Figure 3A), and Egypt (SMD = 1.31, 95% CI = 0.91–1.71, *p* < 0.00001; Figure 3A) in epilepsy patients were higher than those in the control group.

### 3.4 Subgroup analysis by age category

Further subgroup analysis was conducted according to the age of the patients. In adult patients, (df = 5, *p* < 0.00001; *I*^2^ = 99%), the serum MMP-9 level was significantly higher (SMD = 4.44, 95% CI = 1.94–6.93, *p* = 0.0005, Figure 3B) in the experimental group than in the control group. Two studies reported that the serum MMP-9 level in children with epilepsy was not significantly different from that in the control group (*p* = 0.09, Figure 3B). This finding indicates a significant association between serum MMP-9 level and epilepsy in adult.

### 3.5 Subgroup analysis by seizure types

Different seizure types were also compared. Three of the eight studies that analyzed serum samples mentioned the serum MMP-9 level in patients with epilepsy (patients who have been seizure-free for at least 7 days). After the combined analysis, substantial difference in MMP-9 levels can be observed between patients with seizure-free epilepsy (SMD = 2.65, 95% CI = 0.66–4.65, *p* = 0.009; Figure 3C). Five of the eight studies that analyzed serum samples mentioned the serum MMP-9 level in patients with epileptic seizures (including generalized seizures, focal seizures, bilateral tonic–clonic seizures, etc.). The meta-analysis after pooling the data (df = 4, *p* < 0.00001; *I*^2^ = 99%) indicated that the serum MMP-9 level in patients with epileptic seizures was significantly higher than that in the control group (SMD = 5.19, 95% CI = 1.45–8.92, *p* = 0.006, Figure 3C).

### 3.6 Relationship between duration of seizures and serum MMP-9

Two studies reported serum MMP-9 levels in male and female epilepsy patients and matched controls at 1–3, 24, and 72 h after seizure. Four studies reported serum MMP-9 levels in epilepsy patients and matched controls at 1–3, 24, and 72 h after seizure. The subgroup analysis of different durations of seizures was performed, and the fixed and random-effects models were selected based on the presence of heterogeneity/ The value of *p* for heterogeneity was greater than 0.05 by using a random effects model. The meta-analysis indicated that the serum MMP-9 levels at 1–3, 24, and 72 h after seizure in female (1–3 h: SMD = 4.60, 95% CI = −1.29–10.49, *p* = 0.13; 24 h: SMD = 1.95, 95% CI = −0.44–4.34, *p* = 0.11; 72 h: SMD = 0.74, 95% CI = 0.08–1.39, *p* = 0.03; Figure 3D) and male (1–3 h, SMD = 4.60, 95% CI = −1.29–10.49, *p* = 0.13; 24 h: SMD = 1.95, 95% CI = −0.44–4.34, *p* = 0.11; 72 h: SMD = 0.74, 95% CI = 0.08–1.39, *p* = 0.03; Figures 3D,E) epilepsy patients showed no substantial difference between with the control groups.

The meta-analysis of the four studies indicated that the serum MMP-9 levels at 1–3 and 24 h were significantly higher (1–3 h: *p* < 0.00001; 24 h: SMD = 1.02, 95% CI = 0.70–1.34, *p* < 0.00001, Figure 3F) than those of the matched controls. Meanwhile, the serum MMP-9 level at 72 h was not significantly different from that in the control group (72 h: SMD = 3.05, 95% CI = 0.36–5.73, *p* = 0.03, Figure 3F).

## 4 Discussion

Data suggest that approximately 30% of patients with epilepsy do not achieve satisfactory control of the disease by drug treatment, and the proportion of patients with epileptic drug resistance remains unchanged, despite the introduction of new antiepileptic drugs in the last 2 decades, which have not effectively addressed the issue of drug resistance (Kwan et al., 2010; Golyala and Kwan, 2017). On this basis, ideal biological markers that can help the development of new antiepileptic drugs for patients with drug-resistant epilepsy must be determined. Although our comprehension of the physiopathology of epilepsy is not yet comprehensive, existing evidence points to the significant involvement of neuroinflammation, blood–brain barrier dysfunction, and alternation in synaptic remodeling function in epileptic seizure processes. MMP-9 is a protease that regulates a variety of cellular activities, can facilitate the degradation of extracellular matrix proteins, participate in maintaining the integrity of the BBB, and may be involved in the pathogenesis of seizures (Cudna et al., 2017). Pijet et al. (2018) conducted a study on animal models with traumatic brain injury. They found that the elevated level of MMP-9 induced by a traumatic brain injury is related to the activity of spontaneous seizures in the later stage after a traumatic brain injury. The elevated level of MMP-9 increases the sensitivity of drug-induced seizures and the incidence of spontaneous seizures. The high levels of MMP-9 after a traumatic brain injury can promote late-onset epilepsy (Konopka et al., 2013), which may be related to neuroinflammation, synaptic plasticity, and the destruction of the BBB (Meguid et al., 2018). In addition, studies have shown that MMP-9 may play a role in the development of epileptogenesis as an intermediate mediator of neuroimmune processes (Ragin et al., 2009). For instance, elevated levels of high mobility group box 1 protein (HMGB1) in the neuroimmune process may activate metalloproteinases in circulating immune cells and glial cells, leading to increased cytokine production and recurrent seizures (Wang et al., 2021; Li et al., 2022).

Recent studies have shown that that the serum MMP-9 concentration in epilepsy patients was higher than that in the healthy population, which is consistent with previous reports wherein the serum (Suenaga et al., 2008; Li et al., 2013; Cudna et al., 2017) levels of MMP-9 in epilepsy patients were significantly higher after seizures. Therefore, the concentration of MMP-9 in serum may be used as a peripheral marker of neuronal injury and neuroinflammation in the brain associated with epilepsy.

We reviewed current studies on the relationship between serum MMP-9 level and epilepsy and evaluated the potential of MMP-9 as a marker of epilepsy. A total of 13 studies were eligible for inclusion, with a total of 756 patients and 611 matched controls. Eight studies contained results on total serum MMP-9 levels. The corrected meta-analysis integrated and analyzed eight studies that included a total of 556 patients and 432 matched controls and provided adequate quantitative data to compensate for the small sample sizes of the individual studies, which may lead to insignificant, opposite, or enlarged differences. The meta-analysis that included serum MMP-9 data for all epilepsy patients supported a significant increase in MMP-9 in epilepsy patients compared with the control group (SMD = 4.18, 95% CI = 2.18–6.17, *p* < 0.00001). Begg’s test indicates no publication bias in these eight articles. The results of the heterogeneity test suggested severe heterogeneity among the studies, which affected the validity of the meta-analysis. Therefore, we conducted a sensitivity analysis to eliminate three studies that may have caused heterogeneity. We also conducted a subgroup analysis to investigate factors potentially influencing the results.

The subgroup analysis of country showed that serum MMP-9 levels were higher in Chinese, Polish and Egyptian patients with epilepsy than in controls, with more pronounced in Egypt. This may be attributed to race, the degree of economic development, the natural and social environment of the area, and other residual confounding effects. Additionally, the unstandardized sampling time for epileptic patients in different countries may also be an important confounding factor (Maroso et al., 2010). Moreover, the subgroup analysis of age category indicated that the serum MMP-9 level of adult patients with epilepsy was significantly higher than that of matched controls. By contrast, no significant difference in the serum MMP-9 level can be observed between children with epilepsy and matched controls. This finding illustrates that the serum MMP-9 level of children with epilepsy may have little correlation with the disease. The subgroup analysis of seizure types also indicated high serum MMP-9 levels in patients of seizure-free epilepsy (patients who have been seizure-free for at least 7 days) and those with epileptic seizures compared with the healthy control group. The patient duration of seizure subgroup analysis showed that the serum MMP-9 levels at 1–3, 24, and 72 h after seizure in female and male epilepsy patients showed no substantial difference between with the control group. Furthermore, the serum MMP-9 levels at 1–3 and 24 h were significantly higher than those of the matched controls. Nevertheless, the serum MMP-9 level at 72 h was not significantly different from that in the control group. This finding suggests that the elevated serum MMP-9 levels may be related to seizure duration, independent of sex. The subgroups of relevant categorical variables did not fully explain the source of heterogeneity, though it may be attributed to other residual confounding effects. The unstandardized sampling time following an epileptic seizure could be a significant confounding variable. Epileptic seizures and being seizure-free are important trigger factors for the release of MMP-9, which may blur its status as a biomarker for epilepsy assessment. These standard practices must be incorporated, specifically in biomarker research. In the future, researchers should conduct additional studies using standardized sampling times following epileptic seizures and prioritizing the development of more appropriate sampling plans and schedules.

The meta-analysis have limitations because of the small number and variable quality of the included studies. Most of the data used in this study originated from clinical surveys and may have been impacted by biases related to measurement, selection, and interviews. Additionally, several studies lacked a detailed description of the type of epileptic seizure, and the potential effects of factors, such as high fever or trauma, on the serum MMP-9 levels cannot be ignored. Besides, we did not use age and sex as covariates for exploring the relationship between serum MMP-9 and epilepsy due to the lack of clear criteria for dividing the age group and the absence of sex classification in some studies. Instead, we performed subgroup analyses of age and sex separately. Furthermore, it’s worth mentioning that we overlooked the potential effect of the medications taken by the subjects on serum MMP-9 levels in this meta-analysis since the studies included in the meta-analysis specified that the epilepsy patients had been seizure-free for at least two weeks and had relatively stable conditions, indicating minimal fluctuation in serum MMP-9 levels. However, some of the literature did not clearly state whether the subjects were taking medications. Additionally, the subjects had different types of seizures and were taking different types of epilepsy drugs, which could have varying effects on serum MMP-9 levels.

Despite several limitations, the research was executed under strict inclusion and exclusion criteria and this is the first report on this topic. We gained a clearer understanding of how MMP-9 in serum functions as a biological marker of epilepsy through the subgroup analysis of country, age category, seizure types, and duration of seizures. Nonetheless, there are still some shortcomings that need to be addressed and this study has limitations in terms of the diagnosis and treatment of epilepsy. Therefore, a standardized study with a larger sample size must be conducted to verify the outcomes and establish the precise association between the MMP-9 level and the etiology, diagnosis, prognosis, and seizure status of epilepsy. Our research could potentially contribute to clinical diagnosis and treatment in the future.

## 5 Conclusion

This study presents evidentiary support that levels of MMP-9 are significantly elevated in individuals with epilepsy. The detection of MMP-9 is crucial in the early diagnosis of epilepsy, the evaluation of treatment effectiveness, prognosis, and determination of the key medical interventions.

## Author contributions

QW: Writing – original draft, Writing – review & editing. ZL: Writing – review & editing. CY: Writing – review & editing. JL: Data curation, Investigation, Writing – review & editing. JC: Data curation, Investigation, Writing – review & editing. LD: Methodology, Supervision, Writing – review & editing.

## References

[ref1] Bronisz E. Cudna A. Wierzbicka A. Jopowicz A. Kurkowska-Jastrzebska I. (2018) Correlation of blood-brain barrier markers with disease activity, Epilepsia. S57. Wiley: Hoboken, NJ.

[ref2] Bronisz E. Cudna A. Wierzbicka A. Kurkowska-Jastrzębska I. (2022). Serum proteins associated with blood-brain barrier as potential biomarkers for seizure prediction. Int. J. Mol. Sci. 23:14712. doi: 10.3390/ijms232314712, PMID: 36499038 PMC9740683

[ref3] Bronisz E. Cudna A. Wierzbicka A. Kurkowska-Jastrzębska I. (2023). Blood-brain barrier-associated proteins are elevated in serum of epilepsy patients. Cell 12:368. doi: 10.3390/cells12030368, PMID: 36766708 PMC9913812

[ref4] Bronisz E. Kurkowska-Jastrzębska I. (2016). Matrix metalloproteinase 9 in epilepsy: the role of neuroinflammation in seizure development. Mediat. Inflamm. 2016, 1–14. doi: 10.1155/2016/7369020, PMID: 28104930 PMC5220508

[ref5] Chen Y. Chen X. Liang Y. (2023). Metaanalysis of HMGB1 levels in the cerebrospinal fluid and serum of patients with epilepsy. J. Neurol. Sci. 44, 2329–2337. doi: 10.1007/s10072-023-06720-0, PMID: 36933099

[ref6] Cudna A. Bronisz E. Jopowicz A. Kurkowska-Jastrzębska I. (2023). Changes in serum blood-brain barrier markers after bilateral tonic-clonic seizures. Seizure-Eur. J. Epilep. 106, 129–137. doi: 10.1016/j.seizure.2023.02.012, PMID: 36841062

[ref7] Cudna A. Jopowicz A. Mierzejewski P. Kurkowska-Jastrzębska I. (2017). Serum metalloproteinase 9 levels increase after generalized tonic-clonic seizures. Epilepsy Res. 129, 33–36. doi: 10.1016/j.eplepsyres.2016.11.006, PMID: 27886560

[ref8] Diao L. Haichun Y. Zheng J. ZirongChen D. H. Lu Y. (2015). Abnormalities of the uncinate fasciculus correlate with executive dysfunction in patients with left temporal lobe epilepsy. Magn. Reson. Imaging 33, 544–550. doi: 10.1016/j.mri.2015.02.011, PMID: 25705021

[ref9] Dong X. Song Y. N. Liu W. G. Guo X. L. (2009). Mmp-9, a potential target for cerebral ischemic treatment. Curr. Neuropharmacol. 7, 269–275. doi: 10.2174/157015909790031157, PMID: 20514206 PMC2811860

[ref10] El Moneam Abdallah N. A. Kandeel W. A. Elmalt H. A. Elabdeen M. S. Z. (2019). Evaluation of serum matrix metalloproteinase-9 as a potential biomarker for diagnosis of epilepsy in children. Sci. J. Al-Azhar Med. Faculty Girls 3, 587–595. doi: 10.4103/sjamf.sjamf_57_19

[ref11] Fabene P. F. Laudanna C. Constantin G. (2013). Leukocyte trafficking mechanisms in epilepsy. Mol. Immunol. 55, 100–104. doi: 10.1016/j.molimm.2012.12.009, PMID: 23351392

[ref12] Fabene P. F. Mora G. N. Martinello M. Rossi B. Ottoboni L. Bach S.. (2008). A role for leukocyte-endothelial adhesion mechanisms in epilepsy. Nat. Med. 14, 1377–1383. doi: 10.1038/nm.1878, PMID: 19029985 PMC2710311

[ref13] Frigerio F. Frasca A. Weissberg I. Parrella S. Friedman A. Vezzani A.. (2012). Long-lasting pro-ictogenic effects induced in vivo by rat brain exposure to serum albumin in the absence of concomitant pathology. Epilepsia 53, 1887–1897. doi: 10.1111/j.1528-1167.2012.03666.x, PMID: 22984896 PMC3651831

[ref14] GBD 2015 Neurological Disorders Collaborator Group (2017). Global, regional, and national burden of neurological disorders during 1990-2015:a systematic analysis for the global burden of disease study 2015. Lancet Neurol. 16, 877–897. doi: 10.1016/S1474-4422(17)30299-5, PMID: 28931491 PMC5641502

[ref15] Golyala A. Kwan P. (2017). Drug development for refractory epilepsy:the past 25 years and beyond. Seizure 44, 147–156. doi: 10.1016/j.seizure.2016.11.022, PMID: 28017578

[ref16] Hirose G. (2013). An overview of epilepsy: its history, classification, pathophysiology and management. Brain Nerve 65, 509–520. PMID: 23667116

[ref17] Huang Q. Liu J. Shi Z. Zhu X. (2020). Correlation of MMP-9 and HMGB1 expression with the cognitive function in patients with epilepsy and factors affecting the prognosis. Cell. Mol. Biol. 66, 39–47. doi: 10.14715/cmb/2020.66.3.6, PMID: 32538745

[ref18] Konopka A. Grajkowska W. Ziemiańska K. Roszkowski M. Daszkiewicz P. Rysz A.. (2013). Matrix metalloproteinase-9 (MMP-9) in human intractable epilepsy caused by focal cortical dysplasia. Epilepsy Res. 104, 45–58. doi: 10.1016/j.eplepsyres.2012.09.018, PMID: 23182966

[ref19] Kwan P. Arzimanoglou A. Berg A. T. Brodie M. J. Allen Hauser W. Mathern G.. (2010). Definition of drug resistant epilepsy: consensus proposal by the ad hoc task force of the ILAE commission on therapeutic strategies. Epilepsia 51, 1069–1077. doi: 10.1111/j.1528-1167.2009.02397.x, PMID: 19889013

[ref20] Li X. L. Wang S. Tang C. Y. Ma H. W. Cheng Z. Z. Zhao M.. (2022). Translocation of high mobility group Box1 from the nucleus to the cytoplasm in depressed patients with epilepsy. ASN Neuro 14:17590914221136662. doi: 10.1177/17590914221136662, PMID: 36383501 PMC9677174

[ref21] Li Y. Wang Z. Zhang B. Zhe X. Wang M. Shi S.. (2013). Disruption of the blood-brain barrier after generalized tonic-clonic seizures correlates with cerebrospinal fluid MMP-9 levels. J. Neuroinflammation 10:80. doi: 10.1186/1742-2094-10-80, PMID: 23829879 PMC3706217

[ref22] Li N. Xiang L. Qinghua T. Hao W. (2020). Correlation of serum matrix metalloproteinase-9, tumor necrosis factor-α and interferon-γlevels with electroencephalogram of children with epilepsy. Chin. Med. Front. J. 12, 90–94.

[ref23] Maroso M. Balosso S. Ravizza T. Liu J. Aronica E. Iyer A. M.. (2010). Toll-like receptor 4 and high-mobility group box-1 are involved in ictogenesis and can be targeted to reduce seizures. Nat. Med. 16, 413–419. doi: 10.1038/nm.2127, PMID: 20348922

[ref24] Meguid N. A. Samir H. Bjørklund G. Anwar M. Hashish A. Koura F.. (2018). Altered S100 calcium-binding protein B and matrix metallopeptidase 9 as biomarkers of mesial temporal lobe epilepsy with hippocampus sclerosis. J. Mol. Neurosci. 66, 482–491. doi: 10.1007/s12031-018-1164-5, PMID: 30343368

[ref25] Michaluk P. Kaczmarek L. (2007). Matrix metalloproteinase-9 in glutamate-dependent adult brain function and dysfunction. Cell Death Differ. 14, 1255–1258. doi: 10.1038/sj.cdd.4402141, PMID: 17431423

[ref26] Pijet B. Stefaniuk M. Kostrzewska-Ksiezyk A. Tsilibary P.-E. Tzinia A. Kaczmarek L. (2018). Elevation of MMP-9 levels promotes Epileptogenesis after traumatic brain injury. Mol. Neurobiol. 55, 9294–9306. doi: 10.1007/s12035-018-1061-5, PMID: 29667129 PMC6208832

[ref27] Ragin A. B. Wu Y. Ochs R. Scheidegger R. Cohen B. A. McArthur J. C.. (2009). Serum matrix metalloproteinase levels correlate with brain injury in human immunodeficiency virus infection. J. Neurovirol. 15, 275–281. doi: 10.1080/13550280902913271, PMID: 19444696 PMC3469319

[ref28] Rempe R. G. Hartz A. M. S. Soldner E. L. B. Sokola B. S. Alluri S. R. Abner E. L.. (2018). Matrix metalloproteinase-mediated blood-brain barrier dysfunction in epilepsy. J. Neurosci. 38, 4301–4315. doi: 10.1523/JNEUROSCI.2751-17.2018, PMID: 29632167 PMC5932641

[ref29] Stang A. (2010). Critical evaluation of the Newcastle-Ottawa scale for the assessment of the quality of nonrandomized studies in meta-analyses. Eur. J. Epidemiol. 25, 603–605. doi: 10.1007/s10654-010-9491-z, PMID: 20652370

[ref30] Suenaga N. Ichiyama T. Kubota M. Isumi H. Tohyama J. Furukawa S. (2008). Roles of matrix metalloproteinase-9 and tissue inhibitors of metalloproteinases 1 in acute encephalopathy following prolonged febrile seizures. J. Neurol. Sci. 266, 126–130. doi: 10.1016/j.jns.2007.09.011, PMID: 17928006

[ref31] Swann J. W. Hablitz J. J. (2000). Cellular abnormalities and synaptic plasticity in seizure disorders of the immature nervous system. Ment. Retard. Dev. Disabil. Res. Rev. 6, 258–267. doi: 10.1002/1098-2779(2000)6:4<258::AID-MRDD5>3.0.CO;2-H, PMID: 11107191

[ref32] Tao H. Gong Y. Yu Q. Zhou H. Liu Y. (2020). Elevated serum matrix Metalloproteinase-9, Interleukin-6, hypersensitive C-reactive protein, and homocysteine levels in patients with epilepsy. J. Interf Cytok Res. 40, 152–158. doi: 10.1089/jir.2019.0137, PMID: 31971845

[ref33] Vezzani A. Balosso S. Ravizza T. (2008). The role of cytokines in the pathophysiology of epilepsy. Brain Behav. Immun. 22, 797–803. doi: 10.1016/j.bbi.2008.03.009, PMID: 18495419

[ref34] Wang S. Correlation between serum MMP-9 level and cognitive function in patients with epilepsy. Hebei: Hebei Medical University. (2019). 10.3969/j.issn.1673-4254.2017.04.21 PMC6744104 28446411

[ref35] Wang S. Guan Y. Li T. (2021). The potential therapeutic role of the HMGB1-TLR pathway in epilepsy. Curr. Drug Targets 22, 171–182. doi: 10.2174/1389450121999200729150443, PMID: 32729417

[ref36] Yin P. Yang L. Zhou H. Y. Sun R. P. (2011). Matrix metalloproteinase-9 may be a potential therapeutic target in epilepsy. Med. Hypotheses 76, 184–186. doi: 10.1016/j.mehy.2010.09.013, PMID: 20888701

[ref37] Zhang M. Study on the correlation between MMP-9, TNF- α, IL-6 in serum and EEG of epileptic children and its significance. Zhengzhou: Zhengzhou University. (2019).

[ref38] Zhang C. Hao Y. (2009). Dynamic changes and significance of serum matrix metalloproteinase-9 levels in children with epilepsy. Heilongjiang Med. Sci. 32, 34–35.

[ref39] Zhang Q. Wang X. Ding J. Peng W. Fan F. Dynamic changes of serum MMP-9 in acute stage of GTC seizure in epileptic patients and its relationship with seizure severity preliminary study. Proceedings of the 5th CAAE International Epilepsy Forum, China Anti-Epilepsy Association. (2013).

[ref40] Zhen H. (2014). Epidemiologic study of epilepsy. Chin. J. Modern Neurol. Dis. 14:919.

